# Clinical efficacy and safety of modified Sijunzi decoction for the treatment of ulcerative colitis: A protocol for a systematic review and meta-analysis

**DOI:** 10.1097/MD.0000000000023703

**Published:** 2021-01-29

**Authors:** Shu Wei Tian, Yan Ling Zhang, Bin Wang, Ji Ping Liu, Chuan Wang, Juan Zhang

**Affiliations:** College of Pharmacy, Shaanxi University of Chinese Medicine, Xianyang, China.

**Keywords:** meta-analysis, modified Sijunzi decoction, protocol, systematic review, ulcerative colitis

## Abstract

**Background:**

Ulcerative colitis (UC) is a chronic non-specific intestinal inflammatory disease with unknown etiology. In recent years, the global incidence has been increasing. Sijunzi decoction (SJZD) is a traditional Chinese medicine that has been used for treatment of other diseases in previous studies as it has no side effects and it has a pharmacological effect in gastrointestinal function, immune system, ulcers, and tissue repair.

**Methods:**

PubMed, Embase, Cochrane Library, GeenMedical, China National Knowledge Infrastructure, Chinese Sci-tech Journals full-text Database, Chinese Biomedical Database, and Chinese Science Citation Database were searched to screen the related literatures of “ulcerative colitis” and “Jiawei Sijunzi decoction”. The research data extracted from above studies was analyzed by Review Manager 5.3 and Stata14.2 software.

**Results:**

This systematic review and meta-analysis will evaluate the efficacy and safety of Jiawei SJZD in the treatment of UC and provide effective evidence for clinical use.

**Conclusion:**

In this study, the published evidence of modified SJZD in the treatment of UC was systematically summarized and evaluated, so that it can be better applied in clinic.

**INPLASY registration number:**

INPLASY2020100102

## 1 Introduction

Ulcerative colitis (UC) is a nonspecific intestinal inflammatory disease with unknown etiology that is mainly characterized by diarrhea, abdominal pain, mucus pus, and bloody stool. Although the exact cause of UC remains undetermined, the condition appears to be related to a combination of genetic and environmental factors. In recent years, the incidence of UC is rapidly increasing worldwide, especially in many developing countries.^[1–4]^ With the deepening of research and the development of treatment level, the cure rate of UC has been improved, but there are still many postoperative complications, or recurrence after drug withdrawal, and the recovery state of the patients is not ideal.

UC is analyzed from the point of view of traditional Chinese medicine (TCM), which belongs to the category of “dysentery’” and “diarrhea”. According to the consensus of TCM diagnosis and treatment experts,^[5]^ UC is caused by weakness of spleen and stomach, dyskinesia, internal stagnation of dampness, injury of liver for a long time, and disturbance of intestinal peristalsis, It can be seen that TCM believes that spleen qi deficiency is the root cause of UC, and throughout, strengthening qi and invigorating the spleen is the first choice for the treatment of UC.^[6]^

Sijunzi decoction (SJZD) is a TCM which is composed of Ginseng, *Atractylodes*, Poria, and Licorice. It has the effects of invigorating qi, replenishing qi and invigorating the spleen. It mainly treats the syndrome of spleen and stomach qi deficiency, and is often used clinically for peptic ulcers and other patients with spleen and stomach deficiency. Ginseng has the effect of invigorating the spleen and stomach; Licorice has the effects of nourishing the spleen and stomach, replenishing qi, and rejuvenating the pulse; *A macrocephala* can invigorate the spleen and qi; Fuling can condense the heart and strengthen the spleen.^[7,8]^ In recent years, many researchers have used SJZD and modified SJZD to treat UC, and found that it has significant therapeutic effect and less adverse reactions, and is more easily accepted by patients. Modern pharmacological studies have shown that modified SJZD has a therapeutic effect by improving mucosal barrier function, improving intestinal flora, promoting the repair of ulcerated mucosa, improving mucosal immune function and slowing down the degree of apoptosis.^[9]^ However, the evidence-based medicine study of modified SJZD in the treatment of UC has not been reported. Therefore, we put forward a set of systematic evaluation scheme to evaluate the efficacy and safety of modified SJZD in the treatment of UC.

## 2 Materials and programs

### 2.1 Data sources and retrieval strategies

#### 2.1.1 Data sources

The 2 searchers searched PubMed, Embase, Cochrane Library, GeenMedical, the China National Knowledge Infrastructure, VIP database, Chinese Biomedical Database and Chinese Science Citation Database by computer.

#### 2.1.2 Retrieval strategies

The title or abstract combines the following keywords and corresponding terms: modified SJZD, UC, randomized controlled trials; details of the search strategy are as follows:

1. #1 (“Ulcerative colitis” [tile/abstract]) or (“UC” [tile/abstract])
2. #2 (“Modified Sijunzi Decoction” [tile/abstract]) or (“Sijunzi Decoction” [tile/abstract])
3. #3 (“Randomized, controlled trial” [tile/abstract]) or (“Clinical controlled trials” [tile/abstract])
4. #1 and #2 and #3

### 2.2 Inclusion criteria

#### 2.2.1 Type of study

This study includes all clinical studies on the treatment of UC with SJZD or modified SJZD. There are no restrictions on publication countries and languages. The main languages are Chinese and English.

#### 2.2.2 Type of patients

According to the Asia-Pacific UC consensus issued by APAGE and 2010 on inflammatory bowel disease,^[10]^ patients diagnosed as UC have only UC and no other concomitant diseases.

#### 2.2.3 Type of intervention

The patients in the treatment group were only treated with SJZD or modified SJZD, while the patients in the control group were treated with routine treatment or other drugs (such as mesalazine).

#### 2.2.4 Type of outcome measures

Primary outcomes:

(1) clinical effective rate
(2) short-term cure rate

Secondary outcomes:

(1) abdominal pain, abdominal distension, diarrhea, purulent and bloody stool, fever
(2) C reactive protein
(3) hemoglobin
(4) liver function
(5) kidney function
(6) adverse events

### 2.3 Search strategy for the included studies

#### 2.3.1 Study selection

Search through the database according to the corresponding keywords, records that meet the inclusion criteria, studies that do not meet the inclusion criteria or repetitive studies are deleted. Two researchers will read the title and abstract carefully to exclude the research that has nothing to do with the topic, and then read the full text to see if it meets the inclusion criteria. If it does not meet the inclusion criteria, delete it.

#### 2.3.2 Data extraction

**Figure 1:**
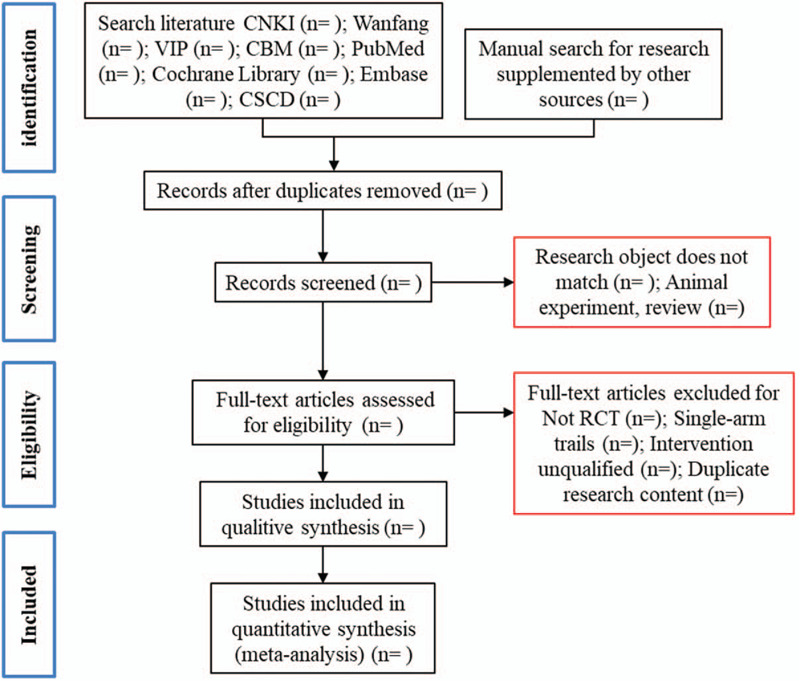
Flow chart for screening qualified studies.

The literature retrieval is independently screened by 2 researchers according to the inclusion and exclusion criteria. If there is a literature with different inclusion, it will be judged by a third party, and the inclusion of the literature will be decided finally after consultation. For the study of data or incomplete data, contact the author for help, if no contact can be made, this document will not be included in the study. Literature research in the screening first read the title and abstract, after excluding the obviously irrelevant research, the rest of the literature will further read the full text to determine whether it is included or not. The 2 researchers independently compiled and recorded the following data:

(i) The 2 researchers independently compiled and recorded the following data
(ii) Patient characteristics (i.e., race, sex, age)
(iii) Outcome indicator data. The details of the study selection will be shown in the PRISMA flow chart (Fig. 1).

#### 2.3.3 Inclusion criteria

According to the literature review and expert suggestions, the selection criteria are as follows:

(i) selected patients must meet the clinical manifestations of UC.
(ii) All trials are randomized controlled trials.
(iii) The experimental group was treated with SJZD or modified SJZD, while the control group was treated with routine treatment or other drugs (such as mesalazine).

The outcome indicators of each study include at least the following 2 indicators: clinical efficacy, recent cure rate, abdominal distension, hematochezia and fever, diarrhea, C-reactive protein, hemoglobin, liver function, kidney function.

#### 2.3.4 Exclusion criteria

The exclusion criteria are as follows:

(i) the patient has other concomitant symptoms.
(ii) The experiment was not described as a randomized controlled trial.
(iii) The outcome indicators of the study did not include clinical effectiveness, short-term cure rate, abdominal pain, abdominal distension.

#### 2.3.5 Heterogeneity analysis

The total clinical effective rate and the recent cure rate are binary variables, expressed by odds ratio, and described by 95% confidence interval. Abdominal distension, purulent stool, fever, diarrhea, C-reactive protein, hemoglobin, and so on are continuous variables. using weighted mean difference or standardized mean difference as the effect index, and 95% CI for description. The Q test was used for analysis, combined with I^2^ to quantitatively determine the degree of heterogeneity. If *P* > 1, the fixed effect model was used for meta-analysis. Otherwise, the random effect model was used.

### 2.4 Risk of bias

Risk bias was independently assessed by 2 investigators. The Cochrane Handbook suggests 7 considerations for evaluating risk of bias as follow:

(1) random sequence generation
(2) allocate and hide
(3) blind participants and personnel
(4) blindness in result evaluation
(5) incomplete result data
(6) selective reporting of results
(7) other biases

Each consideration was divided into 3 levels for the selected studies: “low risk”, “high risk”, or “unclear”.

### 2.5 Publication bias

The funnel chart is used to evaluate the publication bias, in which the effect is used as the Abscissa and the ordinate as the standard error.

### 2.6 Subgroup analysis

Heterogeneity is 1 of the main factors affecting the stability of meta-analysis; when the heterogeneity is large, we can use subgroup analysis to explore the source of the heterogeneity. We will perform a subgroup analysis from the following points to determine the source of heterogeneity: difference in dosage, people of different skin colors, gender difference, Inclusion of differences in studies quality.

### 2.7 Data analysis

Review Manager 5.3 and STATA 14.2 software were used for the meta statistical analysis of the included studies. It was statistically significant when *P* < .05. The Q test was used for analysis, combined with I^2^ to quantitatively determine the degree of heterogeneity.^[11]^ The results of the meta analysis are presented in the form of a forest map, The vertical midline (i.e., the invalid line) divides the icon into a part that is beneficial to the experimental group and a part that is beneficial to the control group. The weight (expressed as a percentage) represents the influence of all included studies on the overall results of the meta analysis. The higher the percentage weight, the larger the representation box, indicating that the study has a greater impact on the overall results. The weight of a study on the overall results is determined by the sample size of the study and the accuracy of the results, and is expressed by the confidence interval.

## 3 Discussion

At present, inflammatory bowel disease includes Crohn disease and UC, and its pathogenesis is more complicated, mainly due to the interaction of genetic factors, immune factors and infection factors.^[12]^ According to the clinical symptoms of UC, it can be classified as “dysentery” and “intestinal seclusion” in Chinese medicine. The main pathogenesis of the disease is weakness of the spleen and stomach, loss of transportation and transformation, and it will turn into fever and fire over time, and dysentery is generated by damp heat.^[13]^ It can be seen that weakness of the spleen and stomach is the main factor in the onset and pathological changes of UC. The existing research^[14,15]^ shows that Modified SJZD and its modified compound can improve UC in clinic. The main mechanism of SJZD in the treatment of UC is to achieve the therapeutic effect by improving the intestinal mucosal barrier and improving the intestinal flora. The intestinal mucosal barrier refers to the function of intestinal epithelium to separate substances in the intestinal lumen and prevent the invasion of pathogenic antigens. It is composed of mechanical barriers, chemical barriers, immune barriers, and biological barriers.^[16]^ The damage of intestinal mucosa or the abnormality of supporting system is the direct cause of the decline of intestinal barrier function. In UC, the lesion is mainly located in the large intestine mucosa and submucosa. The immune system of the intestinal mucosa is disordered, which leads to the infiltration of a large number of inflammatory cells in the intestine. The massive release of inflammatory factors causes the necrosis and shedding of intestinal epithelial cells, which reduces the intestinal mucosal barrier function and breaks The balance of the intestinal mucosal barrier causes UC.^[17]^ UC is related to the interaction among host heredity, susceptibility, and immune factors of intestinal flora in intestinal microecology. The disorder of intestinal flora can induce the abnormal function of intestinal mucosal barrier and lead to the occurrence of UC. The change of intestinal flora structure is considered to be the initial and persistent factor in the pathogenesis of UC, while SJZD can promote the growth of normal intestinal flora.^[18,19]^

Previous studies have shown that,^[20]^ the clinical therapeutic effect of modified SJZD is better than that of mesalazine, and the side effect of modified SJZD is smaller, the price is better, and it is easier to be accepted by patients. Therefore, we used meta-analysis to evaluate the efficacy and safety of modified SJZD in the treatment of UC. However, due to the difference in quality and dosage, we will need to carry out high-quality, large-sample randomized controlled trials in the future to further study and verify the efficacy of Modified SJZD on UC.

## Author contributions

ShuWei Tian: design, conception, edit, and writing manuscripts. YanLing Zhang: Collect literature; performed analysis and interpretation of data. Juan Zhang: Administrative support; review and editing. Bin Wang: Provides important ideas for the article, Analysis of the data in this article. Ji Ping Liu: Strictly revised the article and provided technical support. Chuan Wang: Final approval of the version to be published, and provided financial support.

**Data curation:** YanLing Zhang.

**Formal analysis:** YanLing Zhang.

**Funding acquisition:** Juan Zhang.

**Software:** Juan Zhang.

**Supervision:** Juan Zhang.

**Writing – original draft:** ShuWei Tian.

**Writing – review & editing:** ShuWei Tian.

## Abbreviations

Abbreviations: SJZD = Sijunzi decoction, TCM = traditional Chinese medicine, UC = ulcerative colitis.

## References

[1] Dignass A Eliakim R Magro F. Second European evidence-based consensus on the diagnosis and management of ulcerative colitis Part 1: definitions and diagnosis. Rev Gastroenterol Mex 2014;79:263–89. 25487134 10.1016/j.rgmx.2014.10.001

[2] da Silva BC Lyra AC Rocha R. Epidemiology, demographic characteristics and prognostic predictors of ulcerative colitis. World J Gastroenterol 2014;20:9458–67. 25071340 10.3748/wjg.v20.i28.9458 PMC4110577

[3] Liu C Li Y Chen Y. Baicalein restores the balance of Th17/Treg cells via aryl hydrocarbon receptor to attenuate colitis. Mediators Inflamm 2020;2020:1–9. 10.1155/2020/5918587 PMC7556074 33082710

[4] Samimi N Sepehrimanesh M Koohi-Hosseinabadi O. The therapeutic effect of shark liver oil in a rat model of acetic acid-induced ulcerative colitis. Evid Based Complement Alternat Med 2020;2020:2419230. 33149751 10.1155/2020/2419230 PMC7603576

[5] Zhang SS Shen H Zheng K. Expert consensus on diagnosis and treatment of ulcerative colitis (2017). Chin J Tradit Med Pharm 2017;32:3585–9.

[6] Zou ML, Huang XY, Chen YL, et al. Discussion on the mechanism of Sijunzi Decoction in treating ulcerative colitis based on network pharmacology and experimental verification. Chin J Chin Mater Med. 1-13.

[7] Jiang S. Clinical observation on treatment of ulcerative colitis (spleen deficiency and liver depression) with Sijunzi decoction and Tongxie Yaofang. J Pract Tradit Intern Med 2020;34:83–6.

[8] Yu W Lu B Zhang H. Effects of the Sijunzi decoction on the immunological function in rats with dextran sulfate-induced ulcerative colitis. Biomed Rep 2016;5:83–6. 27347409 10.3892/br.2016.678 PMC4906636

[9] Ji YF Wang RJ Li XB. Research progress on chemical constituents and pharmacological effects of compound Sijunzi decoction. Chin Tradit Herb Drugs 2016;47:837–43.

[10] Ooi CJ Fock KM Makharia GK. The Asia-Pacific consensus on ulcerative colitis. J Gastroenterol Hepatol 2010;25:453–68. 20370724 10.1111/j.1440-1746.2010.06241.x

[11] Higgins JP Thompson SG. Quantifying heterogeneity in a meta-analysis. Stat Med 2002;21:1539–58. 12111919 10.1002/sim.1186

[12] Salim SY Soderholm JD. Importance of disrupted intestinal barrier in inflammatory bowel diseases. Inflamm Bowel Dis 2011;17:362–81. 20725949 10.1002/ibd.21403

[13] Zou ML Ning X Chen YL. Research progress of Sijunzi decoction in preventing and treating ulcerative colitis through intestinal mucosal barrier. Chin Med Herald 2020;26:134–7.

[14] Xue Y Cao ZQ Wang XY. Observation on therapeutic effect of Sijunzi decoction and tongxieyao recipe modified in treating ulcerative colitis with liver depression and spleen deficiency. Liaoning J Tradit Chin Med 2018;45:2352–5.

[15] Chen W Zheng XB. Clinical effect observation of Sijunzi decoction and tongxieyao prescription on ulcerative colitis of liver stagnation and spleen deficiency. J N Pharm 2017;14:127–8.

[16] Gao X Miao R Tao Y. Effect of montmorillonite powder on intestinal mucosal barrier in children with abdominal Henoch-Schonlein purpura: a randomized controlled study. Medicine (Baltimore) 2018;97:e12577. 30278566 10.1097/MD.0000000000012577 PMC6181592

[17] Wang K Wu LY Dou CZ. Research advance in intestinal mucosal barrier and pathogenesis of Crohn's disease. Gastroenterol Res Pract 2016;2016:9686238. 27651792 10.1155/2016/9686238 PMC5019909

[18] Cao J Cha AS. Study on the regulatory effect of Sijunzi decoction on the intestinal flora of rats with ulcerative colitis. Clin J Tradit Chin Med 2019;31:102–4.

[19] Yan MZ Li ZJ Xie NX. Effect of Sijunzi decoction on intestinal flora in experimental spleen deficiency mice. Chin J Mic 1989;40–3.

[20] Chen JS. Comparison of the effects of Sijunzi decoction and mesalazine in the treatment of ulcerative colitis. Nei Mongol J Tradit Chin Med 2020;39:55–6.

